# Cannabis Extract Has a Positive–Immunostimulating Effect through Proteolytic System and Metabolic Compounds of Honey Bee (*Apis mellifera*) Workers

**DOI:** 10.3390/ani11082190

**Published:** 2021-07-23

**Authors:** Patrycja Skowronek, Łukasz Wójcik, Aneta Strachecka

**Affiliations:** Department of Zoology and Animal Ecology, University of Life Sciences in Lublin, Akademicka 13, 20-950 Lublin, Poland; l.wojcik@up.lublin.pl (Ł.W.); aneta.strachecka@up.lublin.pl (A.S.)

**Keywords:** hemolymph, bees’ resistance, immunity of insect, hemp extract, supplementation of bees, glucose, urea, proteolytic enzymes, biochemical pathway, pollinators, biochemistry

## Abstract

**Simple Summary:**

The aim of our study was to test the immunostimulating effect of a diet with hemp extract on the resistance of the honey bee (*Apis mellifera*). The experiment compared the effect of supplementation between the bees receiving the extract in the form of a mixture with sugar syrup and on the strip with the extract, compared to the bees that had no contact with substance. In order to determine this effect, the biochemical indicators were analyzed: the proteolytic system (proteases, protease inhibitors, total protein concentration) responsible for the fight against pathogens/parasites, biomarkers (ALT, AST, ALP), and the basic components of metabolism (glucose and urea concentrations). Parameters were determined in the hemolymph of 2- and 7-day-old workers. Hemp extracts caused an increase in the protein concentrations. Regardless of the method of administration, proteases decreased. Protease inhibitors increased, except supplementation on strips where the activity decreased. The biomarker activities increased in the control group and workers feeding extract in syrup and decreased in workers supplemented with the extract on strips. The results of the metabolic component were as follows: glucose and urea concentrations indicate that the extract will not adversely affect metabolic changes in the insect’s organism. Hemp extract improves the natural immunity of bees.

**Abstract:**

In the study, we assessed the effect of hemp extract on activities of resistance parameters and the metabolic compound concentration in adult workers’ hemolymph. Bees were divided into the following groups: (1) control group fed with mixture of sugar and water-glycerine solution, (2) experimental group with pure sugar syrup and inside with cotton strips soaked with hemp extract, (3) experimental group with a mixture of sugar syrup with hemp extract. Hemp extracts caused an increase in the protein concentrations and reduced the protease activities regardless of the administration method. The protease inhibitor activities were decreased only in the group that received hemp extract on the strips. The biomarker activities (ALP, ALT, AST) increased from the control group and workers feeding extract in syrup and decreased in workers supplemented with the extract on strips. In young, 2-day-old workers, the glucose concentration was higher in the groups feeding with the extract than in the control. Hemp extract influenced an increase in urea concentrations in workers’ hemolymph in comparison with the control. The hemp supplementation positively influences the immune system of workers, and the appropriate method of administration may be adapted to the health problems of bees.

## 1. Introduction

Entomofauna is one of the most represented groups of living organisms on Earth, and the same organisms are the most endangered. Some insect species have been in close relationships with humans for many centuries. One of these species is the honey bee (*Apis mellifera*) [1]. It is estimated that insects contribute to the pollination of as much as 80% of plants that are food sources. Nowadays, this ecological service plays a key role in providing enough food for the constantly growing human population [2]. For over a dozen years, significant declines in the bee population have been recorded throughout the world. In order to minimize these effects, beekeepers decide to use medicinal or preventive substances to stimulate the bees’ organism against possible dangers. Therefore, the search for supplements/biostimulants/compounds that will strengthen bees’ immunity began. Many substances and compounds with a unique composition have been tested, such as resveratrol, spirulina, vitamin C, caffeine, propolis, and dietary additives such as yeast, soybean meal, and pollen substitutes [3,4,5,6,7,8,9]. Compounds of plant origin (resveratrol, spirulina, caffeine) and the indicated dietary additives showed a positive effect on the organism of bees, mainly by extending lifespan or in the case of studies conducted by Strachecka [6], additionally stimulation of the activity of the proteolytic system and/or stimulation of the activity of antioxidant enzymes which counteract the aging of the organism was noted [3,4]. In the case of bees fed with spirulina, there was an increase in physical values, i.e., weight or an increase in abdominal lipids and proteins e.g., in the head part [4]. Supplementation with vitamin C increased the activity of the antioxidant system, reducing losses after winter by up to 33% compared to control groups [5]. Other dietary supplements usually led to an increase in protein concentration or extending the lifespan [7,8]. One of such substances may be hemp extract, which, thanks to the content of active substances from the cannabinoid group, is widely used in medicine as an agent that affects the nervous system (epilepsy, multiple sclerosis) and has a high antioxidant potential (supporting the regeneration of the body after exposure to degrading factors) [10,11,12]. Hemp (*Cannabis sativa*) is the most known and widespread species of hemp cultivated for various purposes and used in many industries and environmental protection. The main active compounds include: cannabidiol, cannabichromene, cannabigerol, Δ9-tetrahydrocannabinol, and cannabinol [12,13].

**Scheme 1:**
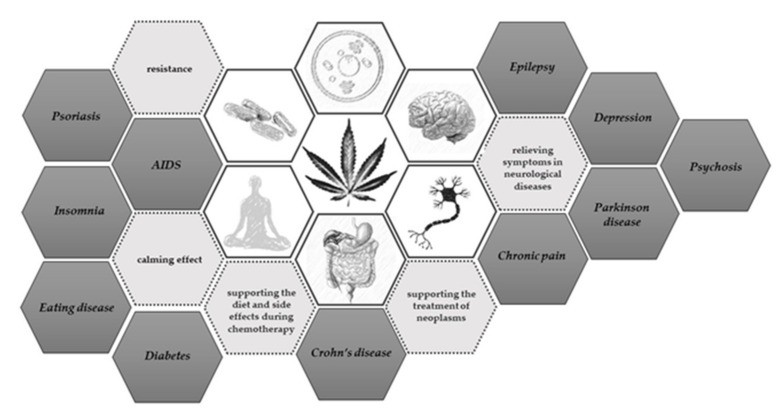
Use of cannabis extract. Hemp in various forms has been tested in the treatment of many neurological, psychological, cancer, viral, and metabolic diseases [11,14,15,16,17].

The extract is mainly used in cases of multiple sclerosis; drug-resistant epilepsy; pain during cancer; phantom, post-traumatic, and chronic pains; and side effects of treatment with chemotherapy/radiotherapy and Alzheimer’s diseases (Scheme 1) [10,11,14].

Its beneficial properties have been tested on other insects, e.g., *Galleria mellonella* [18]. Due to the above-mentioned properties, the extract may prove to be a good solution in solving problems with individual factors of the phenomenon of colony collapse disease—CCD. Therefore, the aim of our research was to determine the influence of hemp extract on the activities of the proteolytic system and enzymatic biomarkers as an element of bee resistance and also their vitality [19,20,21]. The proteolytic system composed of proteases and their inhibitors in the bee’s organism is responsible for inactivating pathogens by destroying their proteins (proteases) or by inhibiting the activity of their proteolytic enzymes (protease inhibitors) acting on immune proteins in insects. Their activity supports bees in their fight against factors that have a negative impact on their health [22,23]. The biomarkers selected for this experiment are intended to help characterize the supplement’s effect by providing information on the level of damage and the severity of apoptosis in key immune tissue cells such as the fat body and hemolymph. The severity of damage is especially visible in older bees, whose tissues are subject to degradation and general inflammatory changes [24,25]. Damage causes the activation and release of specific enzymes to the external bee’s body environment, which is reflected in changes in the biochemical levels of the hemolymph [25,26]. The tested glucose and urea concentrations provide with information on the effect of the supplement on the metabolic changes of sugars (glucose concentration) and proteins (urea concentration) [13]. Additionally, the evolution of beekeeping presents scientists with new challenges. The expanding offer of medicinal substances and methods of their administration forces scientists to carry out more extensive analyses. Therefore, in the experiment we used two methods of administering the extract; the first method consisted of fabric strips, often used in the treatment of varroosis disease, and the other one using a 1:1 sugar syrup mixture with the test substance (hemp extract).

## 2. Materials and Methods

### 2.1. Cages Part

The experiment was carried out with 1-day-old worker bees (*A. mellifera carnica*; collected from an apiary belonging to the University of Life Sciences in Lublin–51°13′31′′ N, 22°38′07′′ E), which were prepared according to the method of Strachecka [27]. Bees were settled into 30 wooden cages (40 bees/cage) with a volume of 576 cm^3^ each (12 × 12 × 4 cm^3^, length/height/width), with a sliding front window, air vents located on the side, and a feeder (a syringe with a modified adaptor) on the top. Optimal conditions were guaranteed in an air-conditioned chamber-constant temperature at 35 °C and 65% relative humidity.

The cages were divided into the following groups:

(1) control group fed with mixture of sugar and water-glycerine solution in a 1:1 ratio ad libidum
(2) experimental group with 1:1 pure sugar syrup ad libidum and inside with cotton strips soaked with 3 mL hemp extract (0.25 g pure hemp paste extract +3 mL water-glycerine solution)
(3) experimental group with a mixture of sugar syrup 1:1 with hemp extract ad libidum (500 mL water-glycerine solution with 4.38 g hemp paste extract)

The water-glycerine solution used in this examination is from Chempur, no. of standards: BN-76/6193-12. The hemp extract was sourced from the manufacturer Melissa.

**Scheme 2:**
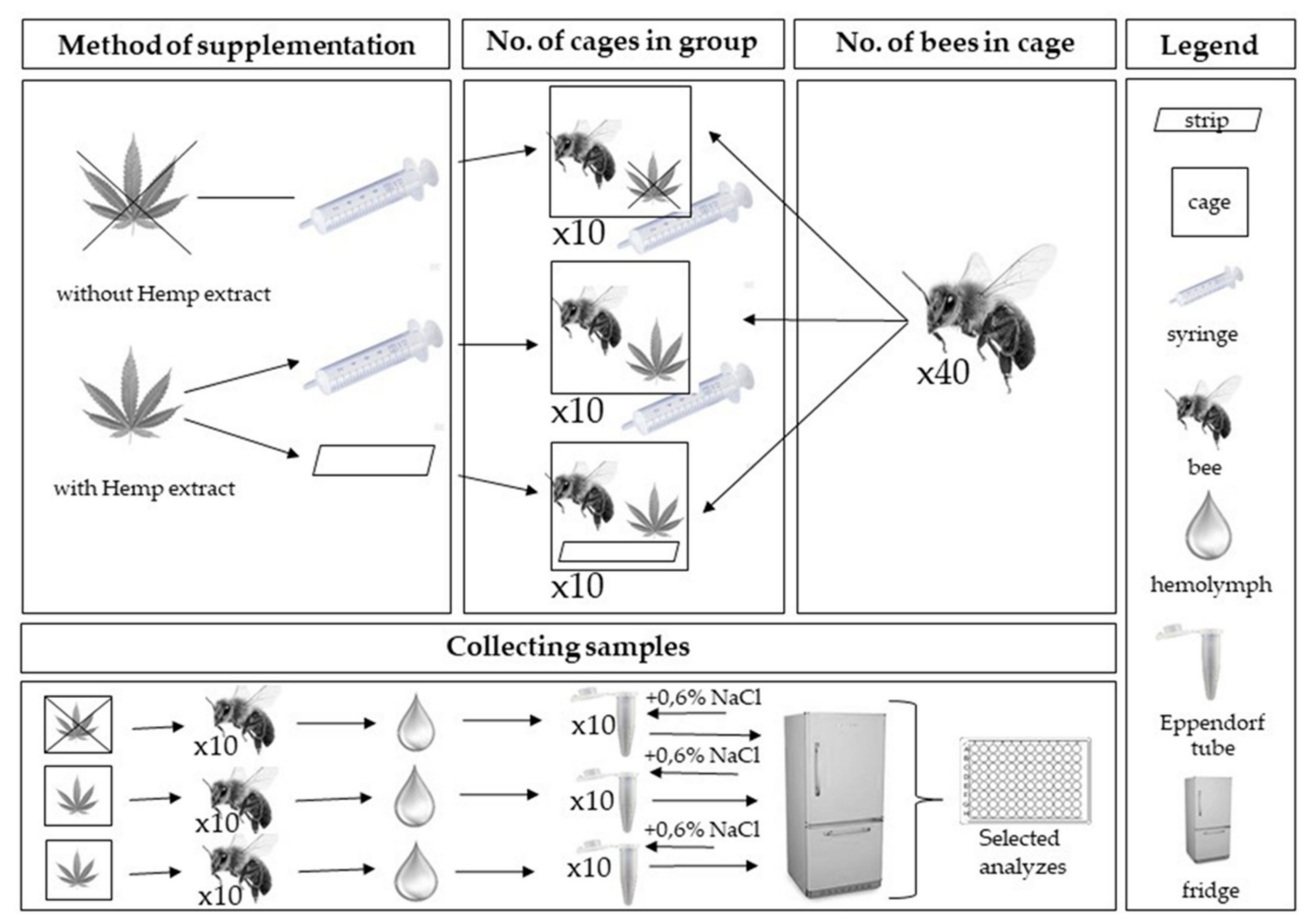
Scheme of collecting samples in experiment.

For each group, 10 randomly selected cages were allocated. In each of the three groups, workers from cages were used for biochemical analyses (Scheme 2). The procedures described below were started 2 days after start of worker feeding (1-day-old bees).

### 2.2. Analytical Part

In each of the groups, fresh hemolymph was taken (puncturing the venous sinus in the insect abdomen into a glass capillary) twice from 10 living workers at age 2- and 7-day [25]. Hemolymph from each bee was separately placed in a sterile Eppendorf tube (hemolymph from one bee = one Eppendorf tube) containing 200 µL of ice-cooled 0.6% NaCl. The samples (3 groups × 2 sampling × 10 workers) were immediately refrigerated at −25 °C for further biochemical analyses. For more details, refer to the Strachecka method [6,28].

The following biochemical analyses were performed in the hemolymph from individual samples:

(1) Total protein concentration was determined using the Lowry method, as modified by Schacterle [29,30];
(2) Proteolytic system activity was determined as follows:
  ■ activities of acidic, neutral, and alkaline proteases according to the Anson method modified by Strachecka and in Łoś [23,25,31,32];
  ■ activities of natural inhibitors of acidic, neutral, and alkaline proteases according to the Lin method [33];
(3) Activities of aspartate aminotransferase (AST), alanine aminotransferase (ALT), and alkaline phosphatase (ALP) using monotests from Cormay (Lublin, Poland) according to the manufacturer’s procedure (methodological details in: File S1);
(4) Concentration of urea and glucose using monotests from Cormay (Lublin, Poland) according to the manufacturer’s procedure (methodological details in: File S1).

## 3. Statistical Analysis

The results were analyzed using Statistica formulas, version 13.3 (2017) for Windows, StatSoft Inc., Tusla, OK, USA. The mixed-model two-way ANOVA followed by post hoc Tukey HSD tests (*p*  =  0.05) were used to compare the results for each basic immunity system parameter (total protein concentration, protease activities, inhibitor protease activities, biomarker activities, urea and glucose concentration) of honey bee workers depending on the method of administration (strip and syringe) and the day (2 day and 7 day) of supplementation with hemp extract.

## 4. Results

**Figure 1:**
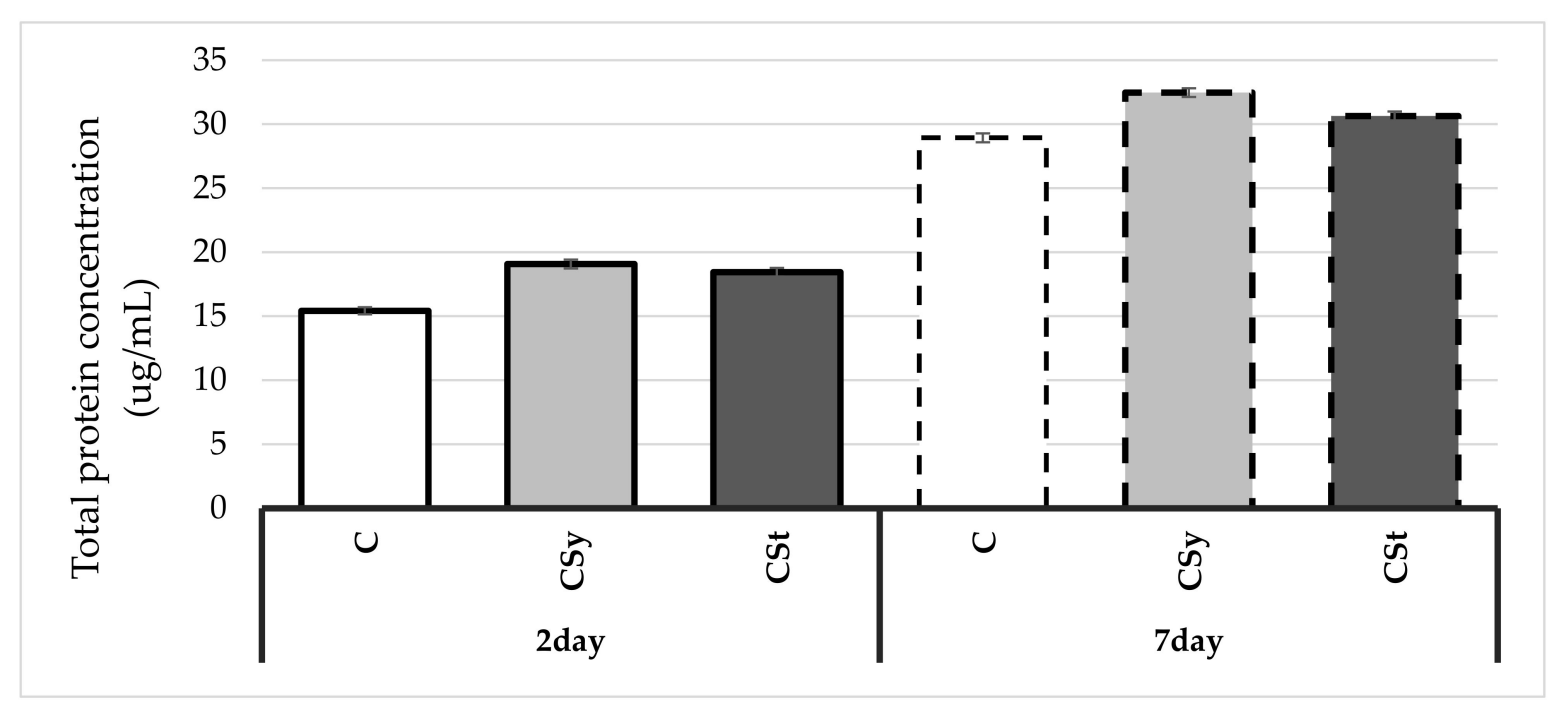
Protein concentrations in the 2- and 7-day-old workers’ hemolymph after two methods of supplementation with hemp extract: C–control (pure sugar syrup), Csy–hemp extract in syrup, Cst–hemp extract on strips (Two-Way ANOVA: supplementation method: F_(2,59)_ = 62.085, *p* = 0.000; days of supplementation: F_(1,59)_ = 2266.3, *p* = 0.000; supplementation method*days of supplementation F_(2,59)_ = 2.2833, *p* = 0.11089).

Protein concentrations increased with age of workers in all groups (Figure 1). Hemp extracts caused an increase in the protein concentrations in the hemolymph of workers. Higher values were always observed in the group that was administered it in the syrup.

**Figure 2:**
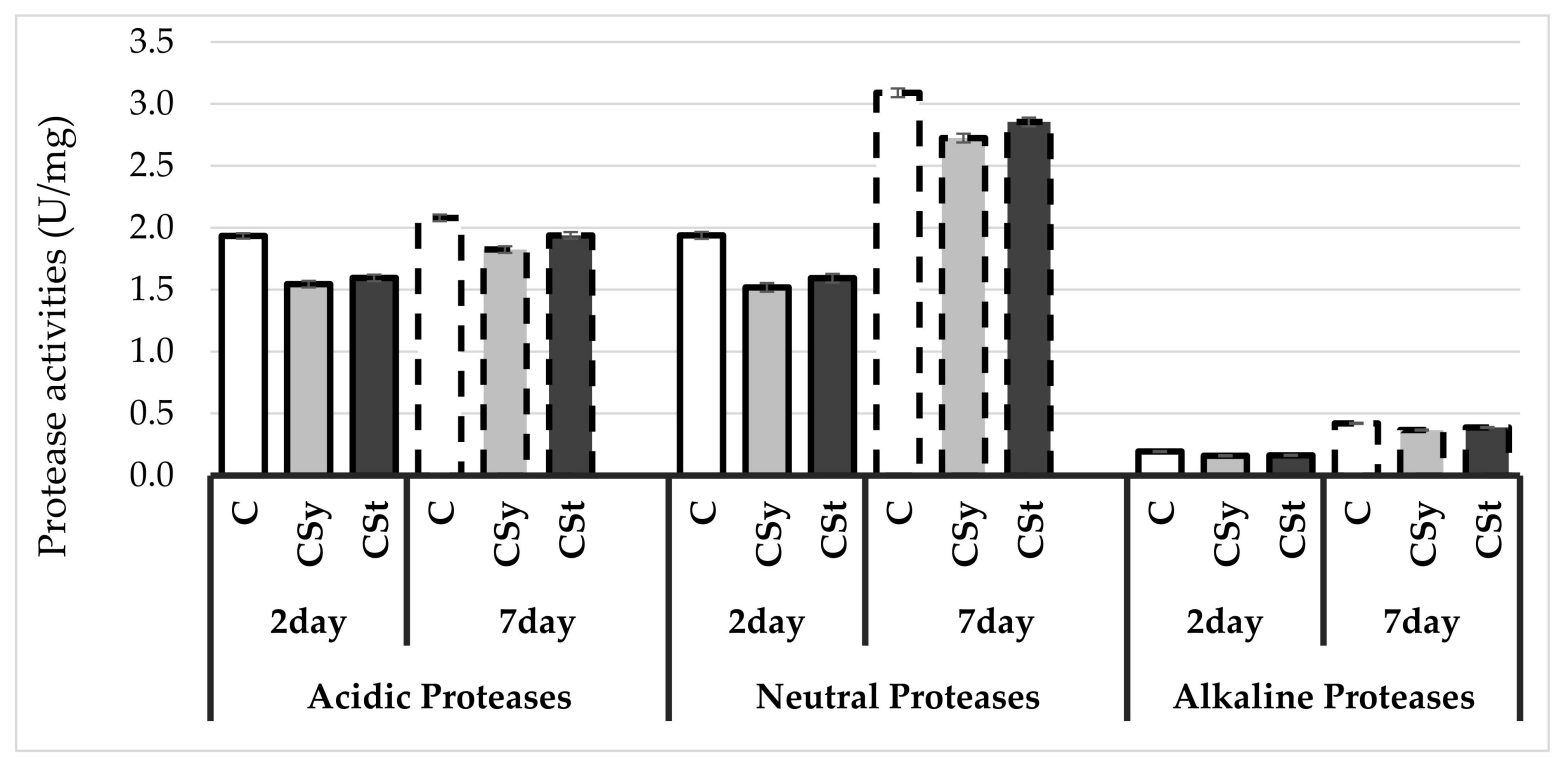
Protease activities in the 2- and 7-day-old honey bees’ hemolymph after two methods of supplementation with hemp extract: C–control (pure sugar syrup), Csy–hemp extract in syrup, Cst–hemp extract on strips (Two-Way ANOVA: Acidic Proteases: supplementation method: F_(2,59)_ = 87.226, *p* = 0.000; days of supplementation: F_(1,59)_ = 145.78, *p* = 0.000; supplementation method*days of supplementation F_(2,59)_ = 7.8591, *p* = 0.00094, Neutral Proteases: supplementation method: F_(2,59) =_ 74.315, *p* = 0.000; days of supplementation: F_(1,59)_ = 1853.4, *p* = 0.000; supplementation method*days of supplementation F_(2,59)_ = 1.3401, *p* = 0.26966, Alkaline Proteases: supplementation method: F_(2,59)_ = 59,780, *p* = 0.000; days of supplementation: F_(1,59)_ = 4008.1, *p* = 0.000; supplementation method*days of supplementation F_(2,59)_ = 3.3549, *p* = 0.04169).

**Figure 3:**
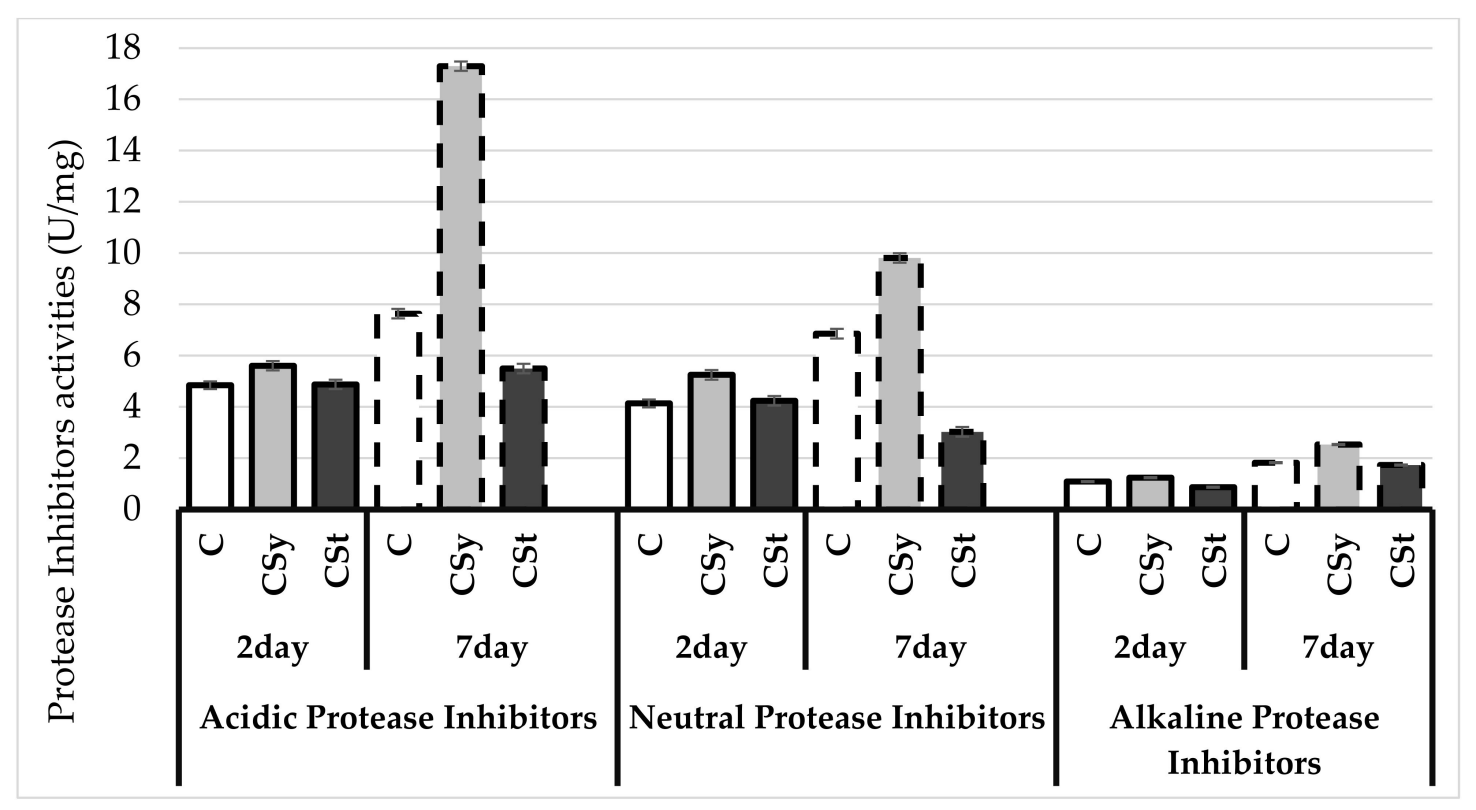
Protease inhibitors activities in the 2- and 7-day-old honey bees’ hemolymph after two methods of supplementation with hemp extract: C–control (pure sugar syrup), Csy–hemp extract in syrup, Cst–hemp extract on strips. (Two-Way ANOVA: Acidic Protease Inhibitors: supplementation method: F_(2,59)_ = 683.41, *p* = 0.000; days of supplementation: F_(1,59)_ = 1203.9, *p* = 0.000; supplementation method*days of supplementation F_(2,59)_ = 521.54, *p* = 0.000, Neutral Protease Inhibitors: supplementation method: F_(2,59)_ = 215.50, *p* = 0.000; days of supplementation: F_(1,59)_ = 184.30, *p* = 0.000; supplementation method*days of supplementation F_(2,59)_ = 124.00, *p* = 0.000, Alkaline Protease Inhibitors: supplementation method: F_(2,59)_ = 404.61, *p* = 0.000; days of supplementation: F_(1,59)_ = 3251.9, *p* = 0.000; supplementation method*days of supplementation F_(2,59)_ = 98.237, *p* = 0.000).

The protease activities as well as acidic and alkaline protease inhibitor activities increased with age of workers (Figure 2 and Figure 3). The extract reduced the protease activities regardless of the administration method. The protease inhibitor activities were decreased only in the group that received hemp extract on the strips. A greater effect of the extract was observed after administration in syrup than in strips compared to the control.

**Figure 4:**
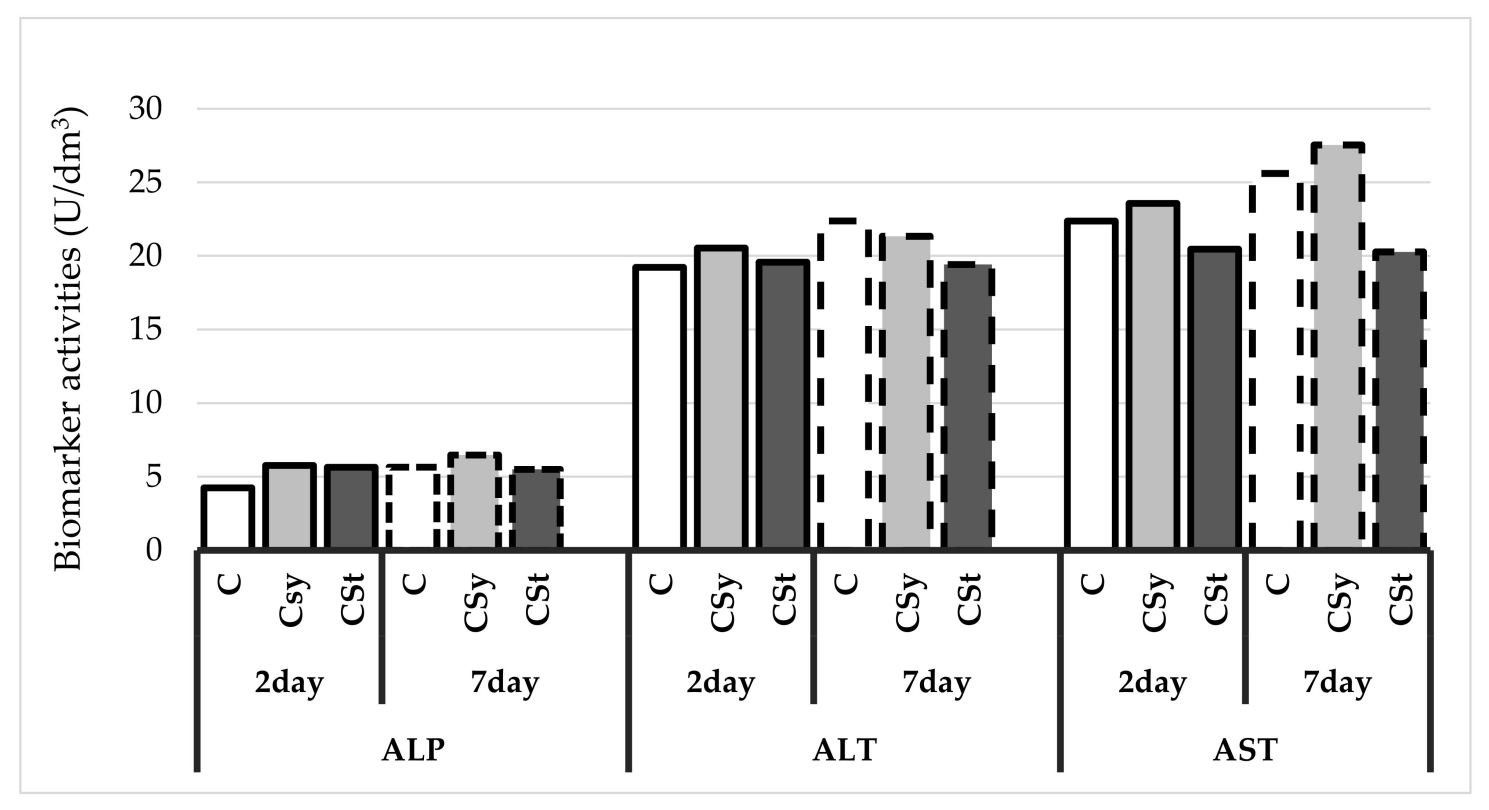
Biomarker activities in the 2- and 7-day-old honey bees’ hemolymph after two methods of supplementation with hemp extract: C–control (pure sugar syrup), Csy–hemp extract in syrup, Cst–hemp extract on strips. (Two-Way ANOVA: ALT (alanine aminotransferase): supplementation method: F_(2,59)_ = 163.68, *p* = 0.000; days of supplementation: F_(1,59)_ = 321.14, *p* = 0.000; supplementation method*days of supplementation F_(2,59)_ = 205.18, *p* = 0,11089, AST (aspartate aminotransferase): supplementation method: F_(2,59)_ = 1180.7, *p* = 0.000; days of supplementation: F_(1,59)_ = 719.49, *p* = 0.000; supplementation method*days of supplementation F_(2,59)_ = 206.94, *p* = 0.000, ALP (alkaline phosphatase): supplementation method: F_(2,59)_ = 204.58, *p* = 0.000; days of supplementation: F_(1,59)_ = 185.72, *p* = 0.000; supplementation method*days of supplementation F_(2,59)_ = 87.550, *p* = 0.000).

The biomarker activities increased with age of workers in the control and in those supplemented with the extract in syrup and decreased in workers supplemented with the extract on strips (Figure 4). Activities of biomarkers were lower in the group with cannabis on strips than other groups.

**Figure 5:**
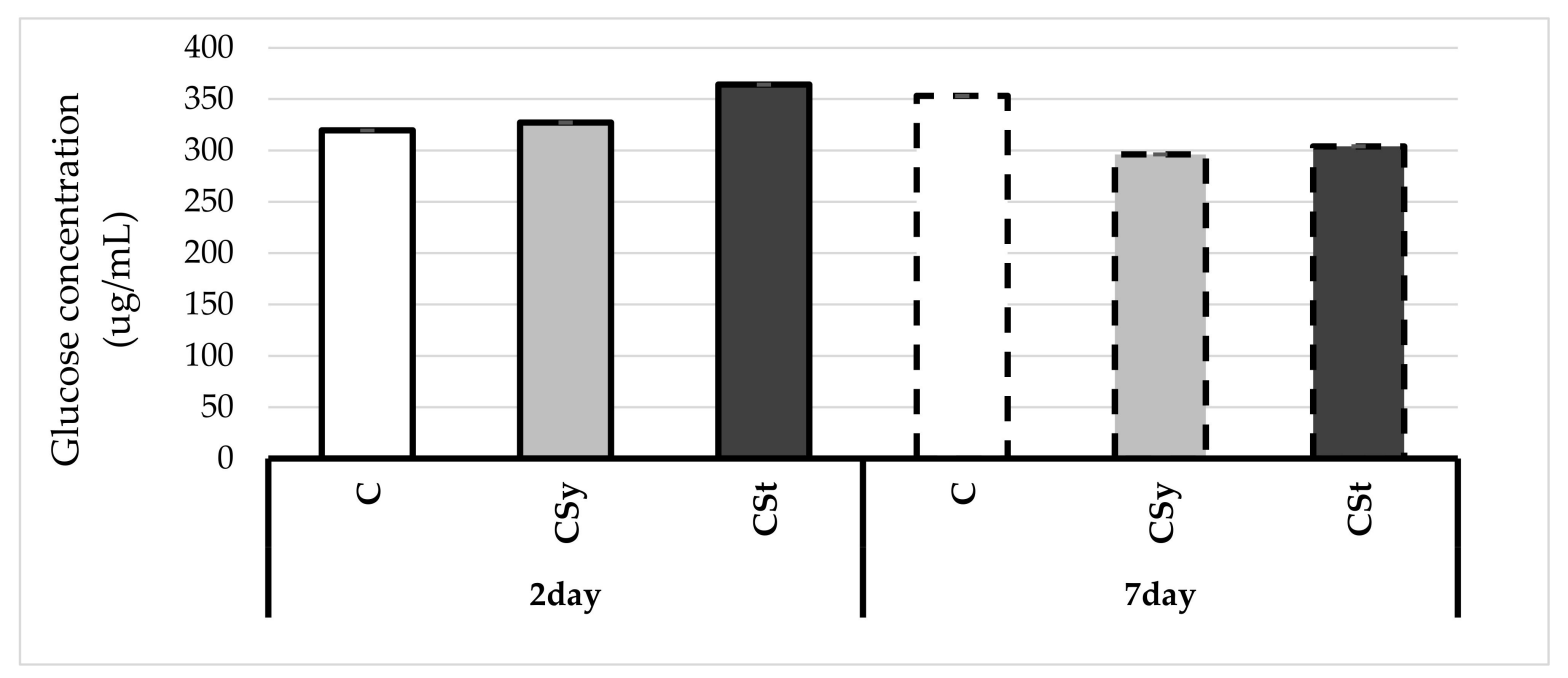
Glucose concentrations in the 2- and 7-day-old workers’ hemolymph after two methods of supplementation with hemp extract: C–control (pure sugar syrup), Csy–hemp extract in syrup, Cst–hemp extract on strips. (Two-Way ANOVA: supplementation method: F_(2,59)_ = 494.15, *p* = 0.000; days of supplementation: F_(1,59)_ = 748.37, *p* = 0.000; supplementation method*days of supplementation F_(2,59)_ = 1639.0, *p* = 0.000).

The glucose concentrations increased with age of workers in the control group, in opposite to the two groups treated with the extract (Figure 5). In young, 2-day-old workers, the glucose concentration was higher in the groups supplemented with the extract than in the control. On the other hand, in 7-day-old workers, the opposite tendency was observed, and the extract decreased the glucose concentration.

**Figure 6:**
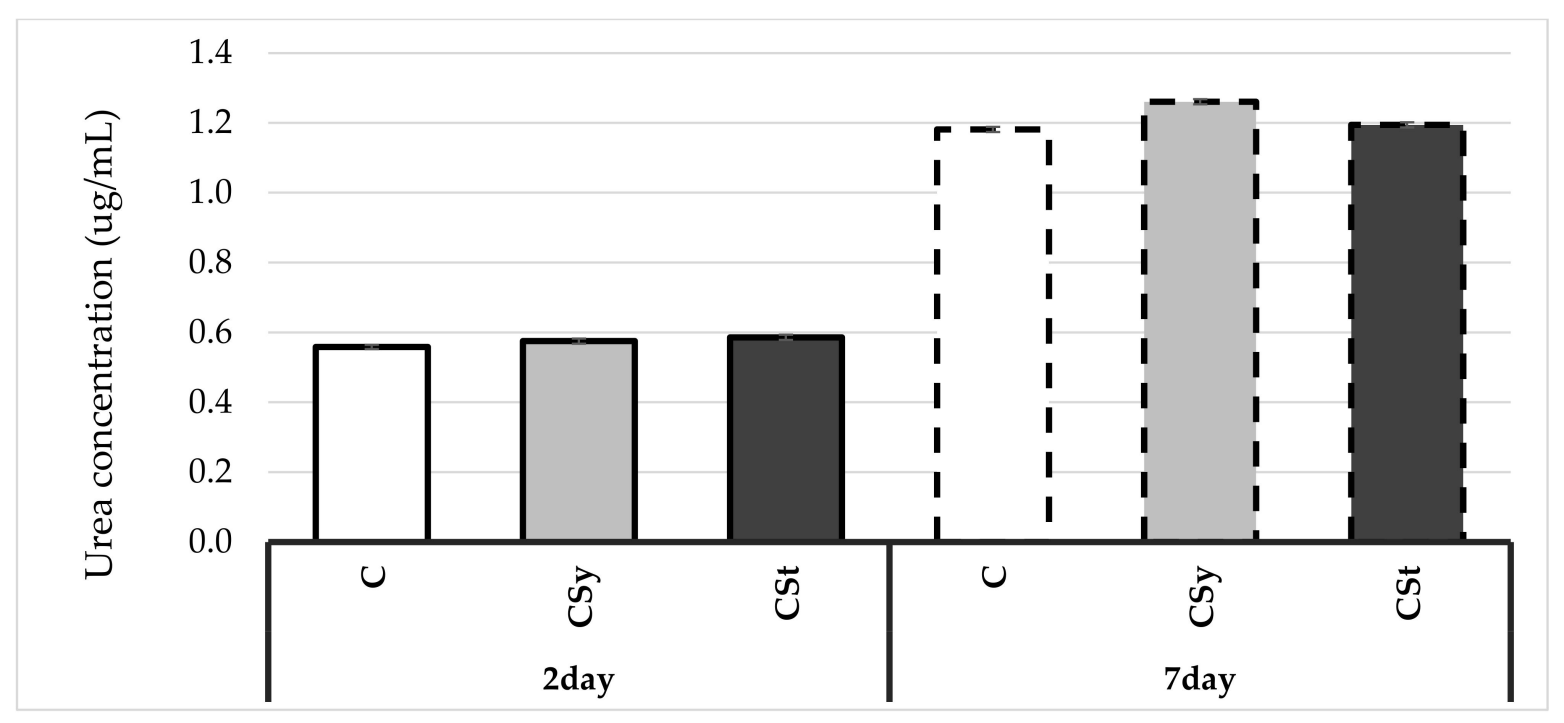
Urea concentrations in the 2- and 7-day-old workers’ hemolymph after two methods of supplementation with hemp extract (Two-Way ANOVA: supplementation method: F_(2,59)_ = 23.004, *p* = 0.000; days of supplementation: F_(1,59)_ = 11912.0, *p* = 0.000; supplementation method*days of supplementation F_(2,59)_ = 15.560, *p* = 0.000). C–control (pure sugar syrup), Csy–hemp extract in syrup, Cst–hemp extract on strips.

Urea concentration increased with age of workers in all groups (Figure 6). Hemp extract influenced an increase in urea concentrations in workers’ hemolymph in comparison with the control group. The highest values were observed in workers supplemented with hemp in syrup.

## 5. Discussion

The positive effect and wide use of hemp extract has been demonstrated in many publications which touched upon neurological, behavioral, and cancer issues [11,34]. Thanks to the scientifically proven properties, we used an extract for bee supplementation in this study. Earlier studies on greater wax moths suggested that giving the supplement to insects living in the close environment of the honey bee will probably be safe also for the pollinators themselves and, as in the experiment with moths, it will have an equally satisfactory/positive effect.

We used two types of supplementation of hemp extracts in the experiment: (1) in sugar syrup (given in syringes), which is the main method in many publications (we can compare the effect of the type of supplementation with others already tested); and (2) with the use of material strips, commonly used in beekeeping practice in the treatment of diseases, e.g., caused by *Varroa destructor* mites. We have decided on these two methods because of their practical aspect in future use as well as in future beekeeping tests. Determining the effect of the supplement depending on the method of administration is important in order to adapt the best methods in the future to the problem of bee colonies. Statistical analysis showed significant changes in the activity of immune parameters depending on the method of administration, which will have an impact on the application of a given method and the activity shown depending on the threats and needs of beekeepers.

The high concentration of total protein for groups fed with hemp extract may indicate their increased production in the fat body, where most of the immune proteins are synthesized. When the extract was fed, the active substances could be absorbed in the digestive system and passed into the hemolymph and surrounding tissues such as the fat body. Some of the active substances in cannabis have been shown to affect the environment inside cells. For example, the aforementioned action, a positive effect of the active substance CBD on the increase in the concentration and homeostasis of Ca2+ ions was noted [35,36]. In immunogenic tissues (fat body, hemolymph), calcium-dependent cellular phospholipases (cPLA2 − Ca + 2-dependent cytosolic phospholipase A2) play a key role in the cellular immunity of insects (e.g., Lepidoptera) [37,38]. Calcium-dependent phospholipases mediate the production of eicosanoids present in the fat body, which are involved in both cellular and humoral immune responses [37]. The effect on humoral immunity may result in an increased production of immune proteins, which translates into a higher total protein concentration reported in our experience. CBD can therefore have an immunostimulating effect. A similar effect was obtained in the studies of Strachecka concerning supplementation with coenzyme Q10 [28], which, similar to CBD, is defined by the authors as a lipophilic antioxidant. In the work of the authors, the concentration of calcium ions was measured, which confirms the potential process indicated by us. In order to confirm this thesis, additional tests should be performed to confirm the concentration of calcium ions in the hemolymph and the fat body of bees.

In addition, hemp extract on the strip could act as an external defensive shield, tightly covering the surfaces of the bees’ body, making it difficult for pathogens to contact with these cuticles. Thus, the substances contained in cannabis could stimulate protein synthesis in the fat body and help seal the surfaces of the insect’s body, in addition to the action of the proteolytic system (proteases and inhibitors presented on a cuticle) [39].

It turned out that the activity of proteases and their inhibitors decreased in the hemolymph of workers who were subjected to hemp extract in experimental groups as compared to the control group.

Alkaline proteases are mostly serine proteases that are involved in many physiological processes in the bee organism; the most important of them is the activation of phenoloxidase, which activates the chinones and tyrosine pathways, and through a number of indirect processes, including the formation of Dopa leads to the activation of sclerotization and melanization mechanisms, as a result of which the bee’s body surface is sealed. Moreover, as a result of melanization, pathogens are coated and locked in melanotic nodules where they are degraded [40].

Our results are in line with the results of Strachecka [6,26], who observed a reduction in the activities of acidic and alkaline proteases after administering curcumin and caffeine to bees ad libitum. The bees, in the experiment with curcumin and caffeine, with similar parameters, showed significantly higher survival than the bees not subjected to supplementation [6,26]. This suggests that the obtained parameters reflect the positive effect on the bee’s organism. A similar result was also obtained in the results of Strachecka on the decrease in the activity of proteases after the use of coenzyme Q10 [28], which, according to the authors’ assumption, also showed a positive effect on the organism of bees. The positive effect of these supplementations can be compared to studies in which potentially negative factors were used. The results of the study by Migdał [41] showed, that the E-field at 50 Hz for 12 h caused an increase in the level of proteases compared to the control group. The authors assumed that the effect of the E-field was negative, suggesting other studies in which the effect of the pesticide bromfenvinphos was tested, after which increased activity of proteases in the hemolymph was observed. In an experiment, Strachecka found that bromfenvinphos significantly lowers the level of biochemical defense of honey bees [27].

The positive effect of the supplementation is confirmed by the above-mentioned studies, in which the activity levels of the parameters in our research are the same as in other tests with factors considered positive (curcumine, coenzyme Q10, caffeine), and different compared to tests with negative factors (E- field, bromfenvimphos).

Lowering the activity of this type of proteases in our results may indicate the previously mentioned sealing of the insect cuticle, reducing the susceptibility to damage, and, as a final result, the alkaline proteases did not need to be activated.

The activities of protease inhibitors in the extract-fed bees were significantly higher than in the control group and the group that received the extract on the strips. This proves the activation of proteolytic reactions, thus strengthening the bee’s organism, which makes it better protected against pathogens. It is worth noting that the activities of the proteolytic system increase with the age of workers, which is consistent with the results obtained by Strachecka [26,28]. Łoś [25] also showed that the activities of proteases and their inhibitors usually increase with the age of workers until about 20–25 days of their life, and then decrease. Bees treated with biostimulants have lower protease inhibitor activities. Protease inhibitors act directly on proteases secreted by pathogens, preventing them from entering body cavities and inhibiting their development. Acid protease inhibitors are directed primarily against pathogenic fungi, basic against bacteria and viruses, and neutral against other agents [19,42]. Based on these studies, it can be concluded that the extract on the strips actually sealed the first immune barriers and limited the penetration of pathogenic factors through these barriers; therefore, the body had no need to respond to the appearance of foreign proteins by producing appropriate enzymes. In the case of the extract in syrup, the bee’s organism strengthened the internal immune mechanisms after being absorbed from the intestine and distributed by the hemolymph throughout the body.

Activities of biomarkers: ALT, AST, ASP were in most cases higher in the bees fed with the hemp extract in the syrup and lower for the bees in group with extract on the strip, compared to the activity in the control bees. Biomarkers determine the functioning, viability, and susceptibility to damage of fat body cells, which is called the “invertebrate liver”. One of the most important biomarkers is the level of ALT. ALT is an intracellular enzyme. Increasing its concentration is often caused by damage to cells that play the role of the liver in insects, i.e., the fat body cells. The lowering of the ALT value on the 7th day of supplementation in both experimental groups proves the potential protection of cells against damage in relation to the body without supplementation. In the case of supplementation on the strips, we observe a reduction in the value of other biomarkers, such as AST, ASP, due to the previously mentioned sealing of the insect cuticle and the creation of an external protective barrier. In the case of supplementation of the extract in syrup, the increased parameters of biomarkers may result from the consumption of a non-standard substance to which the enzymes are not originally adapted and may cause deviations from the norm. In order to determine the further influence of hemp extract on these parameters, their height should be examined in the future in bees older than 7 days. Additionally, the increased activity in relation to the control group should not be negatively considered. The research of Sokol indicates that biomarkers in bees may show the opposite tendency towards mammalian organisms, e.g., have decreased activity during infection with *V. destructor* [43].

CBD (cannabidiol), a substance responsible for sedative effects, has the ability to act intracellularly on mitochondria and nuclear receptors due to cannabidiol lipophilic nature. Hemp extract with syrup may turn out to be more energetic thanks to the influence of active substances on cellular organelles, i.e., mitochondria (the aforementioned effect of CBD), which would result in lower consumption of the syrup and next in lower glucose concentration [35]. In addition, hemp extract, depending on the base (e.g., fat in oils and pastes), may cause changes and a slowdown of sugar metabolism due to the content of fats that digest slower, and as a result the supplied sugar could be metabolized by the body for a longer time [44]. This may be a factor in reducing the glucose concentration in our study. Confirmation of the positive effect of a lower consumption of enriched syrup may be the fact that higher food intake usually occurs in the case of infections, e.g., with microsporidia of *Nosema* spp., where the energy economy is disturbed, and oxidative stress and thermoregulation disorders are undoubtedly a negative effect. Bees suffering from nosemosis consume huge amounts of food [45,46]. This symptom is the opposite reaction to the results obtained in our supplementation.

The increase in urea concentrates in the case of the administration of cannabis in syrup may be related to the increased supply of protein and lipids in the diet of experimental bees compared to control bees, whose main food was sugar. Therefore, we can also observe a slight increase in urea concentration in relation to the control for supplementation with strips due to less direct exposure of bees to the extract compared to administration in syrup. Our results in this case are opposite to the results obtained by Strachecka [26,28]. Coenzyme and curcumin had a lower effect on the level of urea in the bees’ hemolymph, causing an increase in uric acid during the tests. Both substances contain ketone groups which may suggest that the indicated trends are specific for these compounds. Digestion of hemp extract having a different chemical structure compared to the supplementation above may take place in a different way, the effect of which was visible in the increased concentration of urea, and maybe lowered in the case of uric acid. To confirm this, studies should be carried out on the concentration of uric acid in supplementation with hemp extract.

## 6. Conclusions

The hemp supplementation positively influences the immune system of the honey bee, and the appropriate method of administration may be adapted to the health problems of bees. Sealing physiological and anatomical barriers may help in the future with large parasites, such as *V. destructor*, and stimulating the organism from the inside may be helpful in the case of pathogens easily penetrating into organisms such as *Nosema* spp. or also microorganisms, such as bacteria, viruses, and fungi.

## Data Availability

The datasets and materials for this study results that have been used, analyzed and presented in this manuscript are not publicly available. Available on University of Life Sciences in Lublin. At the justified request of the interested party, they may be made available by the respective author.

## Supplementary Materials

The following are available online at https://www.mdpi.com/article/10.3390/ani11082190/s1, File S1: Supplement to the methodology of monotests.
