# Supplementary material for: Cannabis Extract Has a Positive–Immunostimulating Effect through Proteolytic System and Metabolic Compounds of Honey Bee (Apis mellifera) Workers
Source: Animals (Basel). 2021 Jul 23;11(8):2190. doi: 10.3390/ani11082190 (PMC8388424; doi:10.3390/ani11082190)
Supplement: Supplementary file 1 [file animals-11-02190-s001.zip › animals-1270367-supplementary.pdf]

**Alanine Aminotransferase (ALT)**

To determine the ALT activity, a monotest consisting of a one-component reagent was used: 2-ketoglutarate (13mmol/L), L-alanine (440mmol/L), NADH (0.10mmol/L), LDH (1800U/L), Tris buffer (97mmol/L) and EDTA (5.0mmol/L) at pH 7.8. To 100 µl of reagent (37°C) add 10 µl of sample (hemolymph). Reagent-sample mixture mixed on vortex for 3-5 seconds, heated for 30 seconds. at 37 ° C. The absorbance at 340nm was measured during $T_0$, then 1, 2 and 3 minutes after incubation.

**Aspartate Aminotransferase (AST)**

To determine the AST activity, a monotest consisting of a one-component reagent was used: 2-ketoglutarate (13mmol/L), L-aspartate (220mmol/L), LDH (1200U/L), MDH (90U/L), NADH (10mmol/L), Tris buffer (88mmol/L) and EDTA (5.0mmol/L) at pH 8.1. To 100 µl of reagent (37°C) add 10 µl of sample (hemolymph). Reagent-sample mixture mixed on vortex for 3-5 seconds, heated for 30 seconds. at 37 ° C. The absorbance at 340nm was measured during $T_0$, then 1, 2 and 3 minutes after incubation.

**Alkaline Phosphatase (ALP)**

To determine the ALP activity, a test consisting of a component reagent was used: 2-amino-2-methyl-1-propanol (900mmol/L), magnesium acetate (1.6mmol/L), zinc sulphate (0.4mmol/L) and HEDTA (2.0mmol/L). To 100 µl of reagent (37°C) add 2 µl of sample (hemolymph). Reagent-sample mixture mixed on vortex for 3-5 seconds, heated for 30 seconds at 37 ° C. Next, 20 µl 4-NPP (16.0mmol/L) was added to the sample/solution, mixed on vortex for 5 second and heated for 60 seconds. at 37 ° C. The absorbance was measured at 405nm in time $T_0$ and then 1, 2 and 3 minutes after incubation.

Activities of ALT, AST and ALP were calculated according to the formula:

$\text{Activity}_{ALT/AST/ALP} = \Delta\text{Abs/min} \times F$

$F_{ALT/AST} = (TV \times 1000)/(6.3 \times SV \times P)$

$F_{ALP} = (TV \times 1000)/(18.8 \times SV \times P)$

$\Delta\text{Abs/min} = ((A2-A1) + (A3-A2) + (A4-A3))/3$

where:

A1, A2, A3, A4—individual readings of the absorbance values for the samples
TV—total volume of the reaction mixture
SV—sample volume used for the reaction
P—optical path length of the cuvette
6.3—absorbance factor for dihydronicotinamide adenine dinucleotide (NADH; at 340-nm wavelength)

18.8—absorbance factor for 2,4-dinitrophenol (2,4-DNP)

**Glucose**

To determine the glucose concentration, a monotest consisting of a one-component reagent was used: glucose oxidase (> 15,000 U/L), peroxidase (> 1,500 U/L), 4-aminoantipyrine (0.25 mmol/L), phenol (0.75 mmol/L), phosphate buffer (100 mmol/L pH 7 5) and unreactive fillers and stabilizers. To 100 µl of reagent (37°C) add 10 µl of sample (hemolymph). Reagent-sample mixture mixed on vortex for 3-5 seconds, heated for 5min. to 37°C. The absorbance at 500nm was measured during $T_0$.

Concentration of glucose was calculated according to the formula:

Glucose (mg/dl) = $\frac{Abs\_sample}{Abs\_standard}$

where:

Abs – absorbance

**Urea**

To determine the glucose concentration, a monotest consisting of a one-component reagent was used: $\alpha$-ketoglutarate (7,5mmol/L), NADH (>0,20 mmol/L), urease (>5000 U/L), GLDH (>450 U/L), TRIS buffer (100 mmol/L) at pH 8,5. To 100 µl of reagent (37°C) add 10 µl of sample (hemolymph). Reagent-sample mixture mixed on vortex for 3-5 seconds, heated for 5min. to 37°C. The absorbance at 340nm was measured during 30 and 60 seconds after incubation.

Concentration of urea was calculated according to the formula:

Urea = $\frac{\Delta Abs/min.sample}{\Delta Abs/\min catalyst}$

ΔAbs/min = (A2-A1)

where:

A1, A2—individual readings of the absorbance values for the samples
